# Supplementary material for: Tissue resident colonic macrophages persist through acute inflammation and adapt to aid tissue repair
Source: Mucosal Immunol. Author manuscript; Available in PMC 2026 Jun 17. (PMC7619198; doi:10.1016/j.mucimm.2025.11.007)
Supplement: Supplementary Figures [file EMS214250-supplement-Supplementary_Figures.pdf]

Tissue resident colonic macrophages persist through acute inflammation and adapt to aid tissue repair

Lizi M. Hegarty[1,2], Gareth-Rhys Jones[1,4], Adi Biram[3], Claire E. Adams[1], Rebecca M. Gentek[5], Gwo-Tzer Ho[1,4], Elaine Emmerson[2], Calum C. Bain[1,4]

Supplementary Material

Supplementary Figure 1

Supplementary Figure 2

Supplementary Figure 3

Supplementary Figure 4

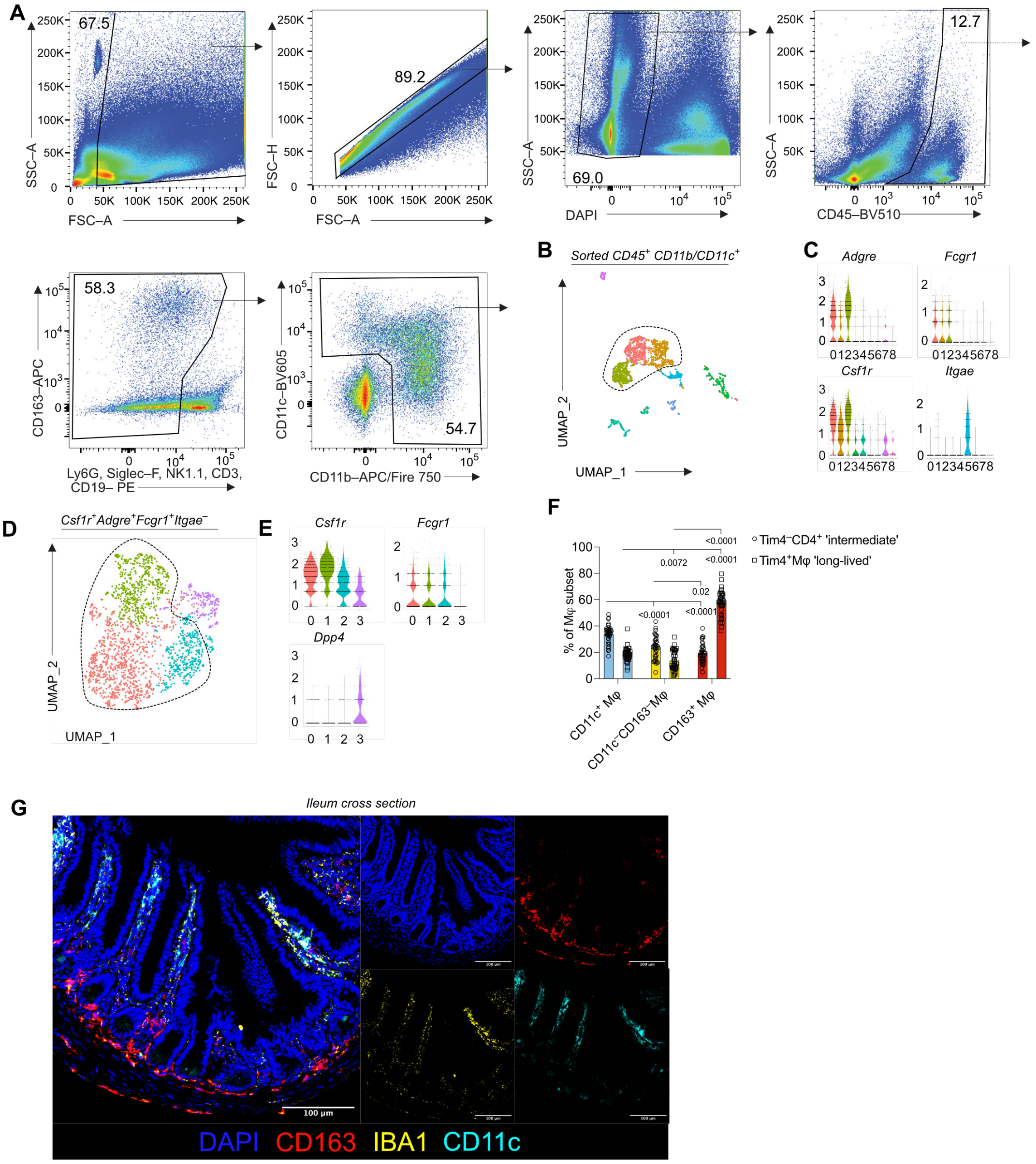

Supplementary Figure 1

A. Gating strategy used for single cell RNA sequencing (scRNAseq): Forward scatter area (FSC−A) vs side scatter area (SSC−A) to exclude debris ; FSC−A vs FSC−height (FSC-H) to exclude doublets/aggregates; SSC−A vs DAPI, live−dead marker to remove dead cells; SSC−A vs CD45 to select CD45+ leukocytes; CD163 vs Ly6G, Siglec−F, NK1.1, CD3 and CD19 to exclude neutrophils, eosinophils, NK cells, T cells, and B cells respectively; CD11c vs CD11b to select mononuclear phagocytes which express CD11c or CD11b.

B. UMAP projection of sorted CD45$^+$ CD11c/CD11b$^+$ colonic cells from naïve adult C57BL/6 mice (as subclustered in Supplementary Fig. 1) and Cells from three mice were pooled for analysis.

C. Violin plots of selected cluster-defining genes from **B.**

D. UMAP projection of *Csf1r/Fcgr1/Adgre1*$^{+}$*Itgae*$^{-}$ subclustered from **B.**

E. Violin plots of selected cluster-defining genes from **D.**

F. Mean frequency of macrophage subset that is Tim4^{-}CD4^{+} (intermediate longevity), and Tim4^{+}CD4^{+} 'long-lived'. Two-way ANOVA with Tukey's post hoc test. n=38 from across 7 individual experimentsin steady-state, C57bl/6J. Symbols represent individual mice. Both male and female mice are included and analysed together.

G. Representative immunofluorescence staining of Hoechst (blue), CD11c (cyan), IBA1 (yellow), CD163 (red) 20μm thick ileum Swiss roll sections.

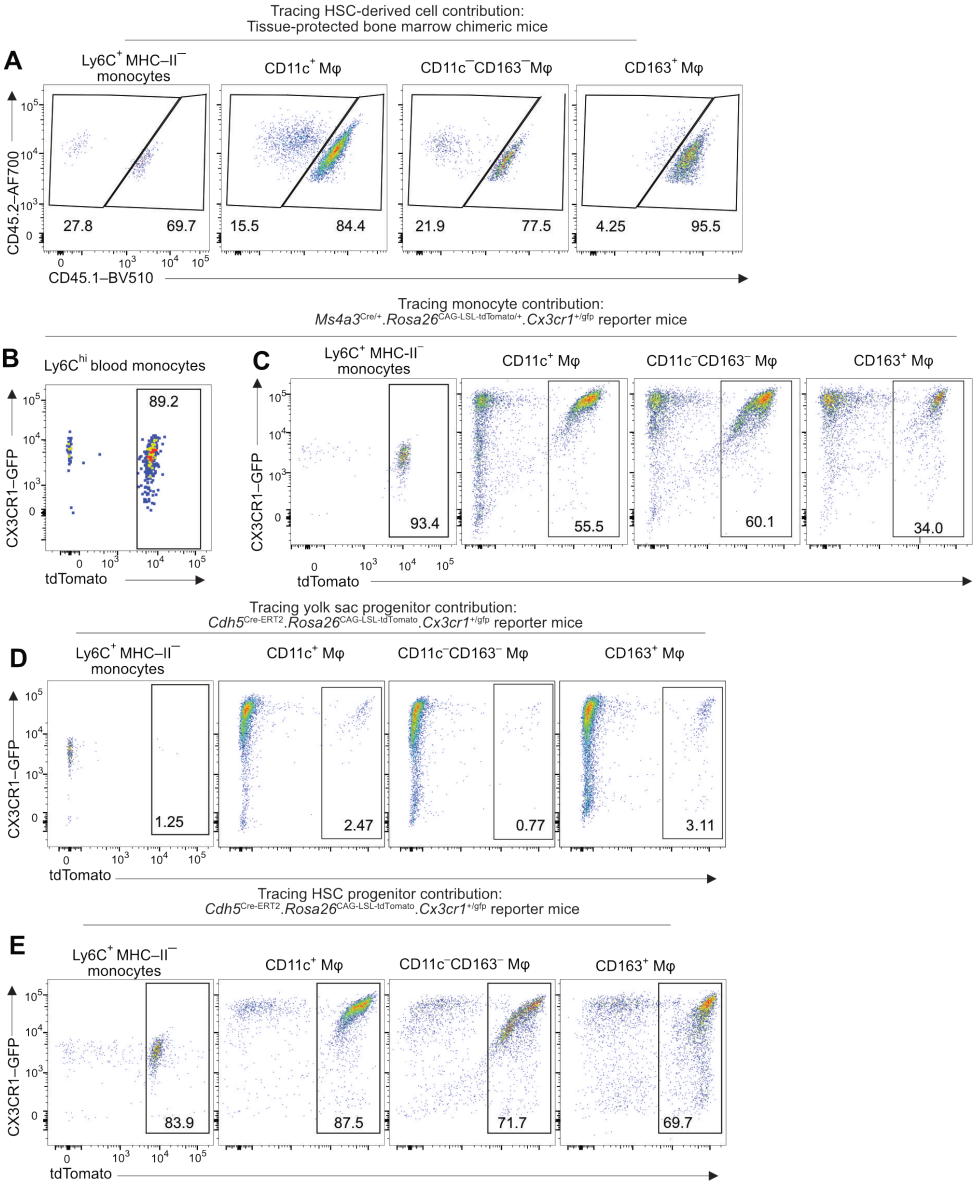

Supplementary Figure 2

A. Representative flow cytometry plots of non-host Ly6C+ MHCII− monocytes, and CD11c+, CD163+, and CD11c−CD163− macrophages from tissue-protected BM chimeric mice, analysed 36 weeks after reconstitution.

B. Representative flow cytometry plot of CX3CR1−GFP and tdTomato expression by blood monocytes
(CD45^{+}CD11b^{+}CD115^{+}Ly6C^{hi}) in Ms4a3$^{Cre/+}$·Rosa26$^{LSL\text{-}CAG\text{-}tdTomato}$ lineage-tracing mice.

C. Representative flow cytometry plots of CX3CR1−GFP and tdTomato expression by Ly6C^{+}MHC-II^{-} monocytes and CD11c^{+}, CD163^{+}, and CD11c^{-}CD163^{-} macrophages in 12-week-old Ms4a3$^{Cre/+}$·Rosa26$^{LSL\text{-}CAG\text{-}tdTomato}$ mice.

D. Representative flow cytometry plots of CX3CR1−GFP and tdTomato expression by colonic Ly6C^{+}MHCII^{-}
monocytes and CD11c^{+}, CD163^{+}, and CD11c^{-}CD163^{-} macrophages in *Cdh5*$^{Cre\text{-}ERT2/+}$.*Rosa26*$^{LSL\text{-}tdTomato/+}$.*Cx3cr1*$^{+/GFP}$ fate mapping mice labelled at E7.5 analysed at 7 weeks of age.

E. Representative flow cytometry plots of CX3CR1−GFP and tdTomato expression by colonic Ly6C^{+}MHCII^{-} monocytes and CD11c^{+}, CD163^{+}, and CD11c^{-}CD163^{-} macrophages in *Cdh5*$^{\text{Cre-ERT2/+}}$.*Rosa26*$^{\text{LSL-tdTomato/+}}$.*Cx3cr1*$^{\text{+/GFP}}$ fate mapping mice labelled at E10.5 analysed at 7 weeks of age.

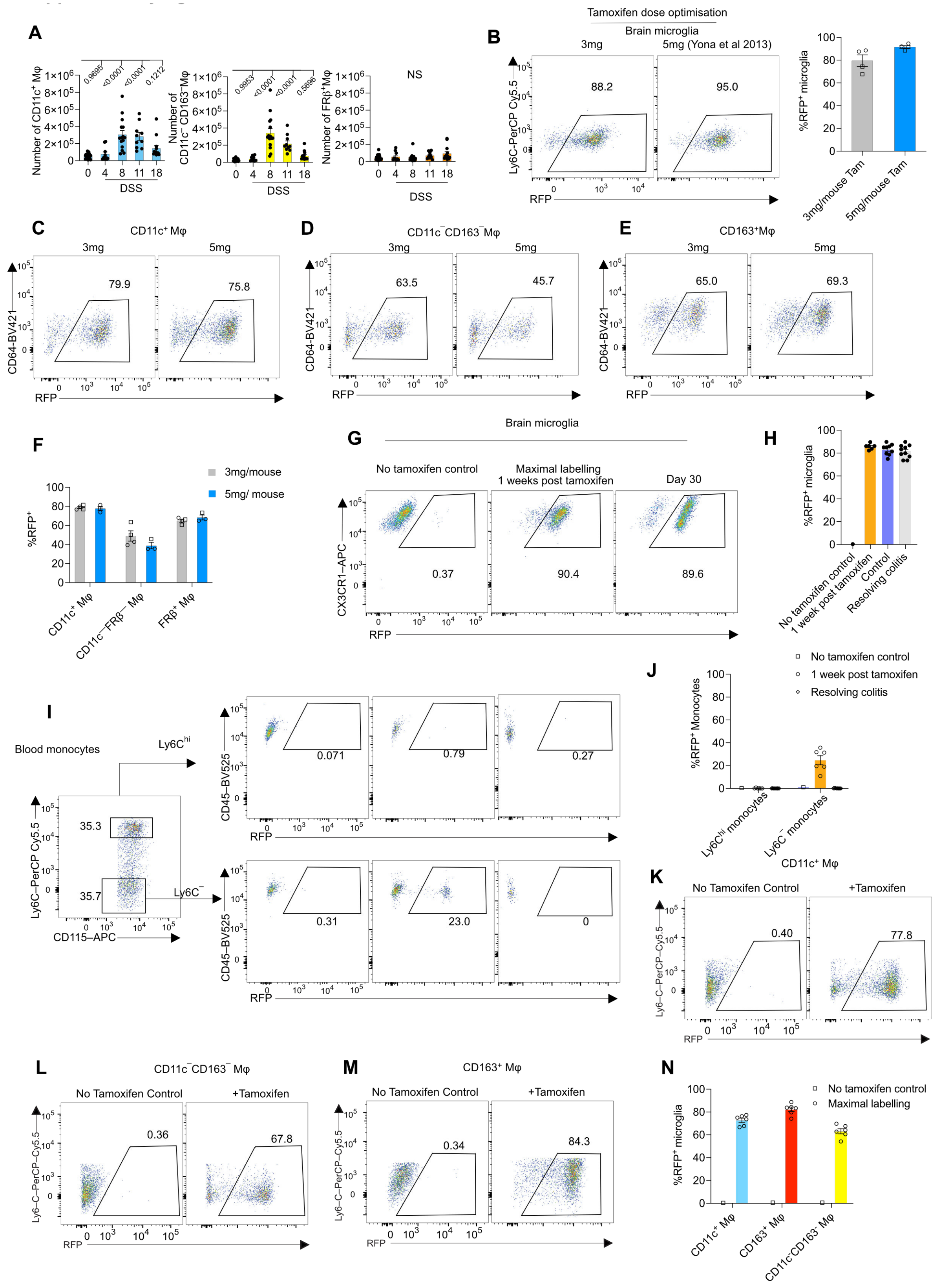

Supplementary Figure 3

A. Mean number of CD11c$^+$, CD11c$^-$ FRβ$^-$, and FRβ$^+$ macrophages across the DSS time course. Data from three independent experiments with n=21 (control mice, day 0), n=15 (day 8), n=14 (day 18). All mice were male. One-way ANOVA followed by Dunnett's multiple comparisons test. Cell numbers are based on FlowJo cell counts, normalised to the numbers of Absolute Counting Beads™ detected per sample. Error bars ± SEM.

B. Representative flow cytometry plot, and mean expression of RFP by brain microglia following 3 or 5 mg tamoxifen administration every day for five days in $\mathit{Cx3cr1}^{\text{Cre-ERT2/+}}.\mathit{Rosa26}^{\text{LSL-RFP/+}}$ mice.

C. Representative flow cytometry plots of RFP expression by $CD11c^{+}$ macrophages following 3 or 5mg tamoxifen administration.

D. Representative flow cytometry plots of RFP expression by $CD11c^{-}$ $CD163^{-}$ macrophages following 3 or 5mg tamoxifen administration.

E. Representative flow cytometry plots of RFP expression by $CD163^{+}$ macrophages following 3 or 5mg tamoxifen administration.

F. Mean RFP expression following 3 or 5mg tamoxifen administration.

G. Representative flow cytometry plots of RFP expression by brain microglia ($CD45^{int}CD11b^{+}MHCII^{-}CX3CR1^{+}$) obtained from $\mathit{Cx3cr1}^{\text{Cre-ERT2/+}}.\mathit{Rosa26}^{\text{LSL-RFP/+}}$ mice one week (maximal labelling) or 29 days post tamoxifen administration, or no tamoxifen controls.

H. Mean RFP expression by brain microglia ($CD45^{int}CD11b^{+}MHCII^{-}CX3CR1^{+}$).

I. Representative flow cytometry plots of RFP expression by blood monocytes ($CD45^{+}CD11b^{+}$ $CD115^{+}$) obtained from $\mathit{Cx3cr1}^{\text{Cre-ERT2/+}}.\mathit{Rosa26}^{\text{LSL-RFP/+}}$ mice one week (maximal labelling) or 29 days post tamoxifen administration, or no tamoxifen controls.

J. Mean expression of RFP by blood monocytes ($CD45^{+}CD11b^{+}$ $CD115^{+}$). Data are pooled from two independent experiments with n=1 (No tamoxifen control), n=6 (Maximal labelling), n=9 (Control) and n=10 (resolving colitis). Both male and female mice were used and analysed together. Symbols represent individual mice. No statistical analysis was performed. Error bars ± SEM.

K. Representative flow cytometry plots of RFP expression by $CD11c^{+}$ macrophages one-week post tamoxifen.

L. Representative flow cytometry plots of RFP expression by $CD11c^{-}$ $CD163^{-}$ macrophages one-week post tamoxifen.

M. Representative flow cytometry plots of RFP expression by $CD163^{+}$ macrophages one-week post tamoxifen.

N. Mean RFP expression. Symbols represent individual mice. Data are pooled from two independent experiments with n=1 (no tamoxifen control), n=6 (maximal labelling). Both male and female mice were used in this experiment. No statistical analysis was performed. Error bars ± SEM

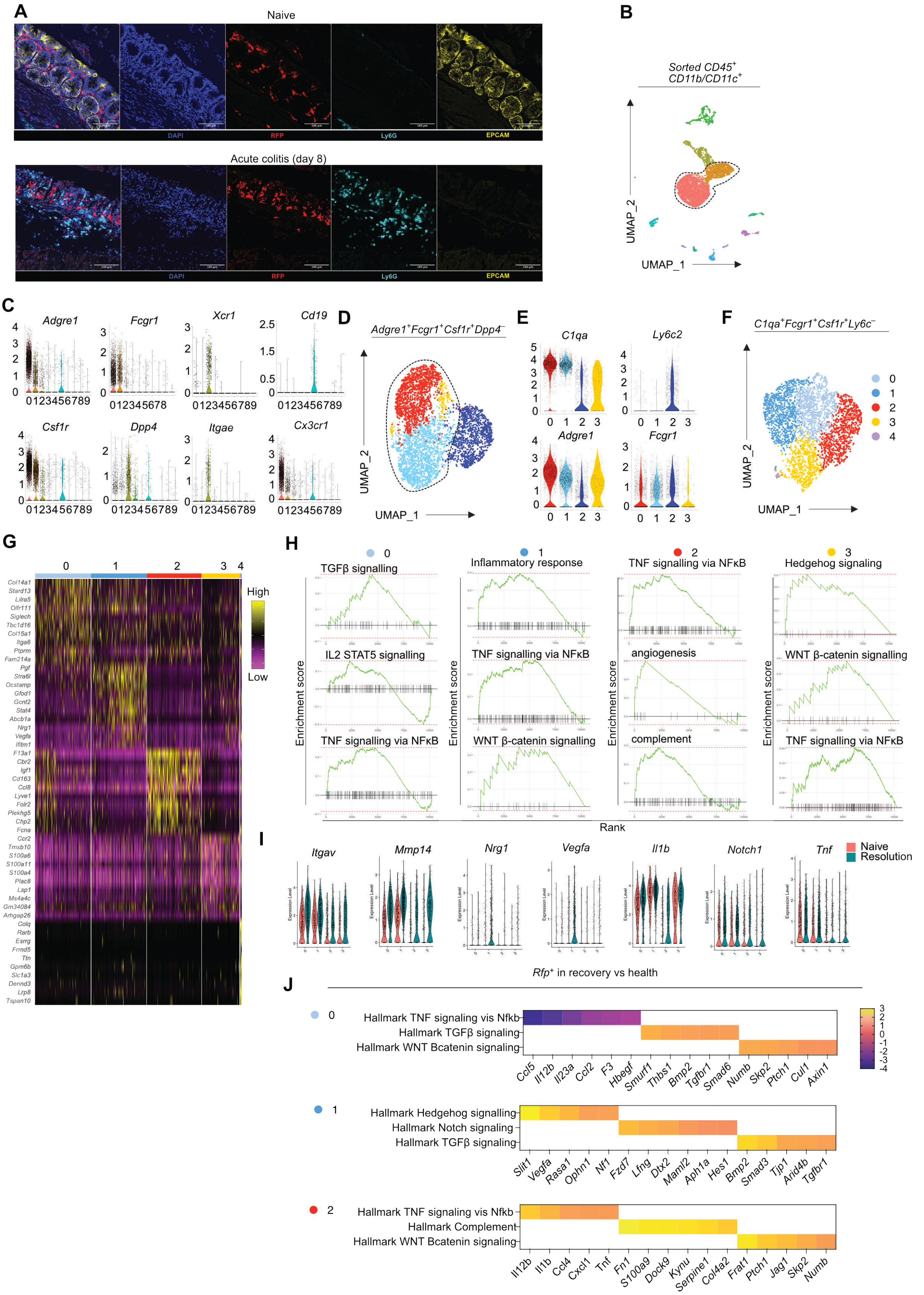

Supplementary Figure 4

a. Immunofluorescence staining of DAPI (blue), RFP (Red; representing fate-mapped macrophages), Ly6G (cyan; marking neutrophils) and EPCAM (yellow; marking epithelial cells) in tamoxifen treated naïve (A) or acutely colitic (B) *Cx3cr1*$^{Cre\text{-}ERT2/+}$.*Rosa26*$^{LSL\text{-}RFP/+}$ mice. 12μm thick Swiss roll sections. Data are from day 8 of the DSS time-course (following 4 days 2%

DSS treatment, and 4 days normal drinking water). Images are representative of n=4 naïve n=5 acutely colitic male and female mice.

B. UMAP projection of sorted CD45^{+} CD11c/CD11b^{+} colonic cells from naïve and resolution *Cx3cr1*$^{\text{Cre-ERT2/+}}$.*Rosa26*$^{\text{LSL-RFP/+}}$ mice mice.

C. Violin plots of selected cluster-defining genes from **B.**

D. UMAP projection of *Csf1r/Fcgr1/Adgre1*$^{+}$*Itgae/Cd19/Xcr1/Dpp4*$^{-}$ cells subclustered from **B.**

E. Violin plots of selected cluster-defining genes from **D.**

F. UMAP (*C1qa*$^{+}$*Ly6c2*$^{-}$) macrophages sub-clustered from **D**.

G. Heatmap displaying the top 10 cluster defining genes for each cluster of macrophages in **F.**

H. Fast gene set enrichment analysis (FGSEA) using Hallmark gene set signatures of each cluster in resolution vs naïve. Enrichment plots.

I Violin plots of selected genes in resolution vs. naive.

J. Fast gene set enrichment analysis (FGSEA) using Hallmark gene set signatures of the *Rfp*$^{+}$ macrophages in each cluster in resolution vs naïve. Heatmap of leading-edge genes for each pathway.
